# Spontaneous Intracranial Hypotension Presenting With Acute Orthostatic Headache: A Case Report

**DOI:** 10.7759/cureus.117240

**Published:** 2026-09-30

**Authors:** Ali Ben Arose, Geetanjali Gupta, Fatima Saeed

**Affiliations:** 1 Acute Medicine, Manchester Royal Infirmary, Manchester, GBR

**Keywords:** csf leakage, migraine, neck pain, orthostatic headache, spontaneous intracranial hypotension

## Abstract

Spontaneous intracranial hypotension (SIH) is an uncommon and frequently under-recognized cause of orthostatic headache, resulting from spontaneous cerebrospinal fluid (CSF) leakage. We report the case of a 39-year-old woman who presented to the Manchester Royal Infirmary hospital with sudden-onset neck pain and headache associated with dizziness, vomiting, and presyncope, markedly worse on standing and relieved by lying flat. The patient's neurological examination was normal; however, she was hypotensive and unable to tolerate an upright posture. Initial computed tomography (CT) and CT angiography of the head were unremarkable. In addition, magnetic resonance imaging (MRI) of the brain and spine with contrast demonstrated diffuse pachymeningeal enhancement and an extensive spinal longitudinal epidural CSF collection extending from the cervical to lumbar spine, consistent with SIH secondary to a spinal CSF leak. She required admission and neurosurgical referral for further investigation and management. This case highlights the importance of recognizing SIH in patients presenting with acute orthostatic headache and neck pain, even in the absence of trauma or focal neurological deficit.

## Introduction

Spontaneous intracranial hypotension (SIH) is a relatively uncommon condition caused by a spinal cerebrospinal fluid (CSF) leak, most often from the spinal dura [1], with an estimated annual incidence of roughly 5 per 100,000 people [2]. It affects women twice as often as men and most frequently strikes middle-aged adults around 40 years old [2]. 

Several causes of SIH have been hypothesized. The most widely accepted theory is the mechanism of a CSF leak. This primary theory describes CSF leak as a disruption of the spinal meninges during a twist or stretch. Dural tear has been identified as the second most common cause of CSF leak from meningeal diverticula, particularly in patients with connective tissue disorders [3]. 

There are a few proposed hypotheses leading to dural tears, such as bony pathology of the cervical spine, especially in patients having posterior osteophytes or microspurs, which are present in the general population. The length of the microspur and narrowing of the spinal canal seem to be the risk factors leading to dural puncture [4-5]. Less commonly, spinal CSF-venous fistulas allow for the spillage of CSF into the circulatory system, inducing CSF hypovolemia [3]. The majority of CSF leaks occur within the spine, specifically the thoracic or cervicothoracic junction. Rarely, CSF leaks can occur at the skull base [3].

A consequence of CSF leak, almost often from the spinal dura, and reduced CSF volume is descent of the brain [6]. This leads to traction or distortion of various anchoring pain-sensitive structures of the brain, causing orthostatic or primarily orthostatic headaches [6]. Venous distention may also play a role in the production of these headaches [6].

The classical presentation is an orthostatic headache, although associated symptoms such as neck pain, nausea, vomiting, dizziness, and visual disturbance are common and may lead to diagnostic confusion [7]. SIH is frequently misdiagnosed as migraine, meningitis, or musculoskeletal pain, resulting in delayed diagnosis [1]. Magnetic resonance imaging (MRI) of the brain and spine plays a key role in identifying the characteristic features such as pachymeningeal enhancement, where the outermost protective layer of the central nervous system, that is, the dura mater, appears bright and thickened, brain sagging, venous sinus engorgement, pituitary enlargement, and subdural collections. In addition, it helps guide further management [8-10]. Early recognition is important, as untreated SIH may lead to complications including subdural collections or hemorrhage [11]. 

The main aim of this case report is to raise awareness of this condition and avoid diagnostic delays in patients presenting with a similar clinical picture. It also highlights an often underdiagnosed and debilitating cause of orthostatic headaches.

## Case presentation

A 39-year-old woman presented with acute-onset neck pain and headache that developed suddenly while getting dressed at home. Earlier that morning, she had felt well. The pain originated in the neck and radiated over the entire head, was moderate in intensity (rated 5-6/10 on the Numerical Pain Rating Scale), and was significantly worse with sitting, standing, or coughing, with partial relief when lying flat.

The headache was associated with marked dizziness, presyncope, blurred vision on standing, and repeated episodes of vomiting. She reported difficulty sitting upright, walking straight, and moving her neck due to pain. She felt generally lethargic and unwell but denied photophobia, fever, rash, seizures, or loss of consciousness. There was no history of recent trauma, falls, lumbar puncture, or surgery. She had no previous history of migraine.

Her medical history was notable for iron-deficiency anemia secondary to menorrhagia. She was not taking regular medications other than intermittent oral iron. She was a nonsmoker and did not drink alcohol.

The main concerning symptom for her was acute neck pain which had radiated to the head. 

On examination, the patient appeared pale and unwell but was alert and orientated. Neck flexion was mildly restricted due to pain, without nuchal rigidity. There was no photophobia or rash. Kernig’s and Brudzinski’s signs were negative.

She was hypotensive on presentation, with a recorded blood pressure of 92/65 mmHg and heart rate of 76 beats per minute, and reported presyncopal symptoms on sitting upright with blood pressure as low as 84/59 mmHg and heart rate of 65 beats per minute, which made us think initially that her symptoms were just a representation of systemic arterial hypotension and anemia. Cardiovascular and respiratory examinations were otherwise unremarkable. Neurological examination revealed normal cranial nerves, full power in all limbs, normal coordination and gait (when tolerated), brisk reflexes, and down-going plantar responses.

Differentials included subarachnoid hemorrhage, postural hypotension, vertebral artery dissection, symptomatic anemia, cervicogenic or musculoskeletal headache, and benign paroxysmal positional vertigo.

Initial blood tests demonstrated significant microcytic anemia consistent with iron deficiency (hemoglobin 83 g/L; low serum iron and transferrin saturation). Inflammatory markers, renal and liver function tests were normal. Pregnancy test, cortisol level, thyroid function, and infectious screening were unremarkable (Table 1).

**Table 1:** Laboratory results Blood results, including thyroid function tests, pregnancy, cortisol, and renal function tests, were all within normal limits. Full blood counts showed features of iron deficiency anemia. WBC: white blood cell; RBC: red blood cell; Hb: hemoglobin; HCG: human chorionic gonadotropin; eGFR: estimated glomerular filtration rate; T4: thyroxine; NBRC: nucleated red blood cell; MCV: mean corpuscular volume; MCH: mean corpuscular hemoglobin; MCHC: mean corpuscular hemoglobin concentration; RDW: red cell distribution width; Plts: platelets; Hct: hematocrit

Laboratory test	Latest reference range and units	Day 1	Day 2	Day 3
WBC	4.0-11.0 × 10^9^/L	4 × 10^9^/L	-	4.5 × 10^9^/L
RBC	3.80-5.50 × 10^12^/L	3.86 × 10^12^/L	-	4.86 × 10^12^/L
Hb	115.0-165.0 g/L	83.0 g/L	-	92.0 g/L
Hct	0.370-0.470 L/L	0.275 L/L	-	0.487 L/L
MCV	80.00-98.00 fL	71.20 fL	-	90 fL
MCH	27.0-33.0 pg	21.5 pg	-	35 pg
MCHC	320-365 g/L	302 g/L	-	340 g/L
RDW	11-15%	19%	-	18%
Plts	150.0-400.0 × 10^9^/L	255 × 10^9^/L	-	228 × 10^9^/L
Neutrophils	1.80-7.50 × 10^9^/L	3.03 × 10^9^/L	-	3.38 × 10^9^/L
Lymphocytes	1.00-4.00 × 10^9^/L	0.67 × 10^9^/L	-	0.79 × 10^9^/L
Monocytes	0.20-1.00 ×10^9^/L	0.26 ×10^9^/L	-	0.29 × 10^9^/L
Eosinophils	0.00-0.40 ×10^9^/L	<0.10 ×10^9^/L	-	<0.10 × 10^9^/L
Basophils	0.00-0.10 × 10^9^/L	<0.10 × 10^9^/L	-	<0.10 × 10^9^/L
Reticulocytes	20-80 × 10^9^/L	39 × 10^9^/L	-	49 × 10^9^/L
Reticulocyte Hb	20.8-30.8 pg	19.3 pg	-	25.2 pg
Immature granulocytes	0.00-2.00 × 10^9^/L	<0.10 × 10^9^/L	-	<0.10 × 10^9^/L
NRBC	0-<0.1 × 10^9^/L	<0.10 × 10^9^/L	-	<0.10 × 10^9^/L
Blood film	-	Red cell morphology consistent with iron deficiency	-	-
Prothrombin time (PT)	9.9-11.8 s	-	11.9 s	-
Activated partial thromboplastin time (APTT)	21-30 s	-	22.5 s	-
Sodium	133-146 mmol/L	140 mmol/L	-	138 mmol/L
Potassium, Bld	3.5-5.3 mmol/L	4.3 mmol/L	-	3.4 mmol/L
Urea	2.5-7.8 mmol/L	4.5 mmol/L	-	2.6 mmol/L
Creatinine	45-84 µmol/L	51 µmol/L	-	54 µmol/L
eGFR	>90 mL/min/1.73 m^2^	>90 mL/min/1.73 m^2^	-	>90 mL/min/1.73 m^2^
Acute kidney injury (AKI)	-	Insufficient data to determine AKI Stage	-	No current indication of AKI
Alanine transaminase (ALT)	1-35 IU/L	9 IU/L	-	-
Alkaline phosphatase (ALP)	30-130 U/L	52 U/L	-	-
Total bilirubin	0-21 µmol/L	9 µmol/L	-	-
Albumin	35-50 g/L	43 g/L	-	-
Globulins	15-42 g/L	33 g/L	-	-
Total protein	60-80 g/L	76 g/L	-	-
C-reactive protein	0-5 mg/L	<1 mg/L	-	<1 mg/L
Serum vitamin B12	197-771 ng/L	358 ng/L	-	-
Folate	3.9-20.0 µg/L	5.5 µg/L	-	-
Iron	5.8-34.5 µmol/L	2.8 µmol/L	-	-
Transferrin	2.00-3.60 g/L	4.05 g/L	-	-
Transferrin saturation	15.0-45.0%	2.80%	-	-
HCG, blood (pregnancy)	0-4 IU/L	<1 IU/L	-	-
Thyroid-stimulating hormone	0.27-4.20 mU/L	-	2.32 mU/L	-
Free T4	11.3-21.6 pmol/L	-	17.1 pmol/L	-
Cortisol	500-800 nmol/L	740 nmol/L	654 nmol/L	-

Computed tomography (CT) of the head and CT angiography showed no acute intracranial pathology (Figures 1-4). Given that CT head had been performed within a six-hour period, subarachnoid hemorrhage was ruled out from the beginning.

**Figure 1:**
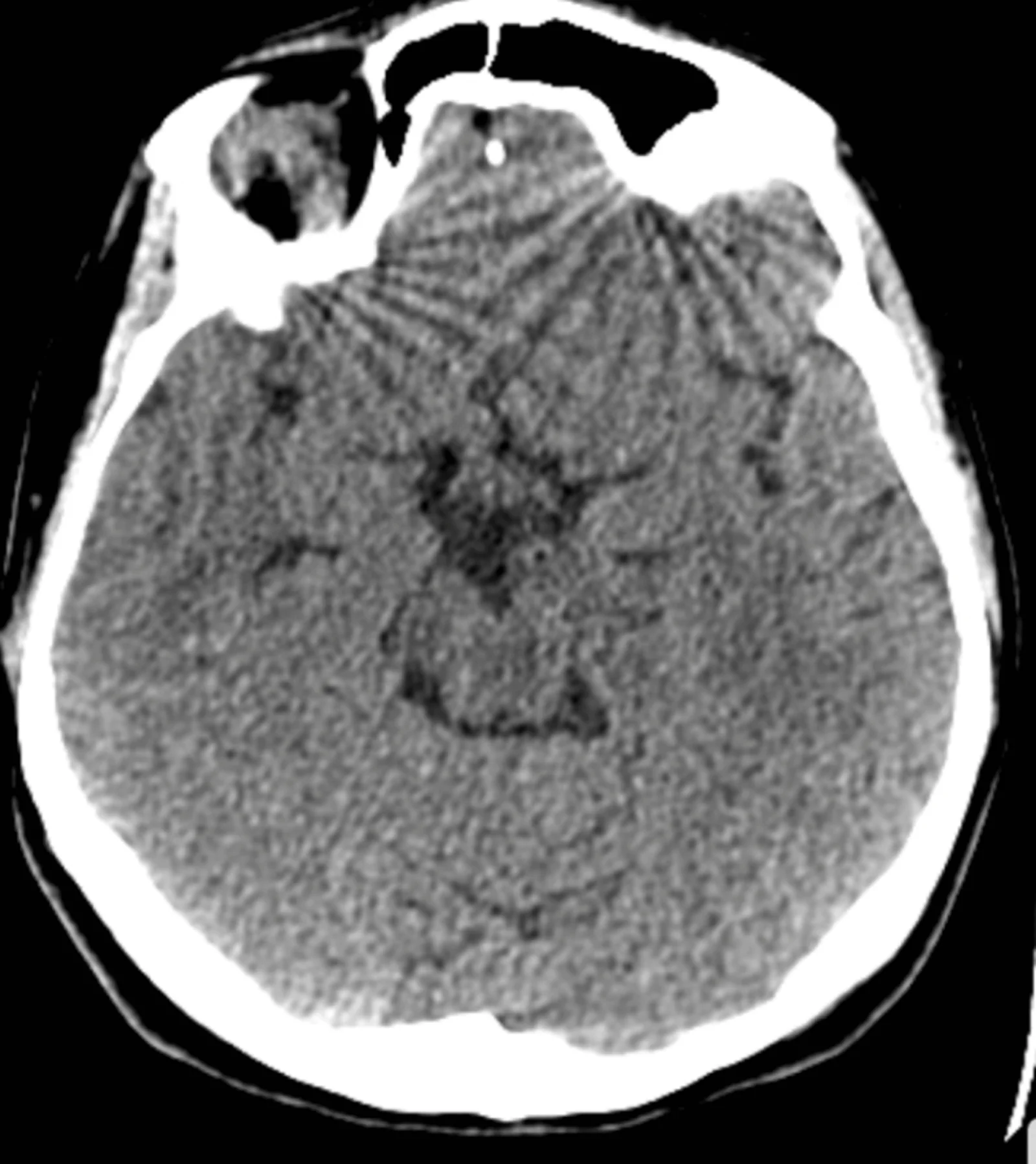
CT head Non-contrast axial CT scan of the head demonstrating normal intracranial anatomy. No evidence of acute hemorrhage, midline shift, or large territorial infarct was seen. The patient presented with worsening gradual headache. CT head was performed to rule out an intracranial event. CT: computed tomography

**Figure 2:**
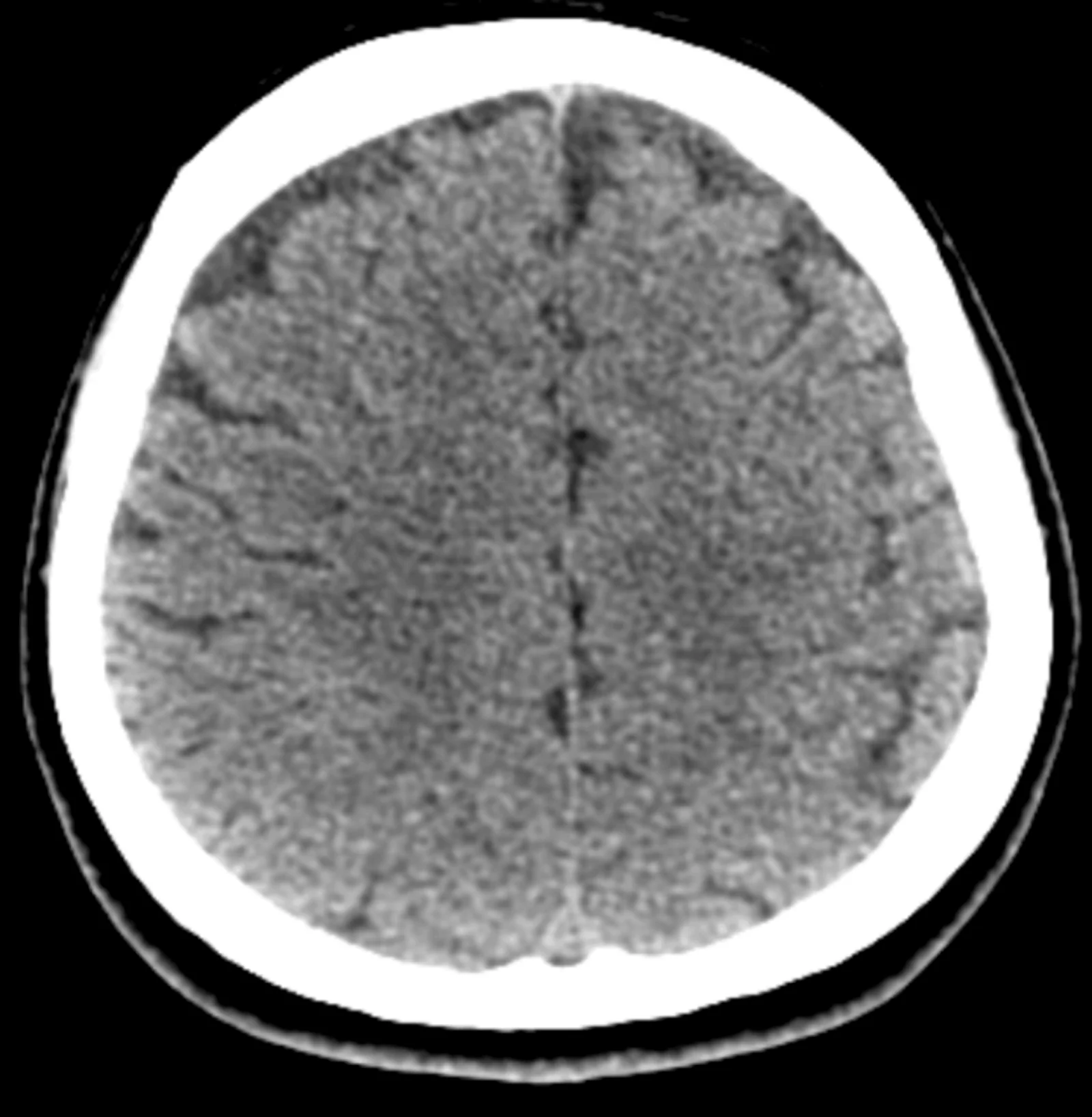
CT head Axial slice of a non-contrast CT head. Sulcal patterns are within normal limits for age. CT: computed tomography

**Figure 3:**
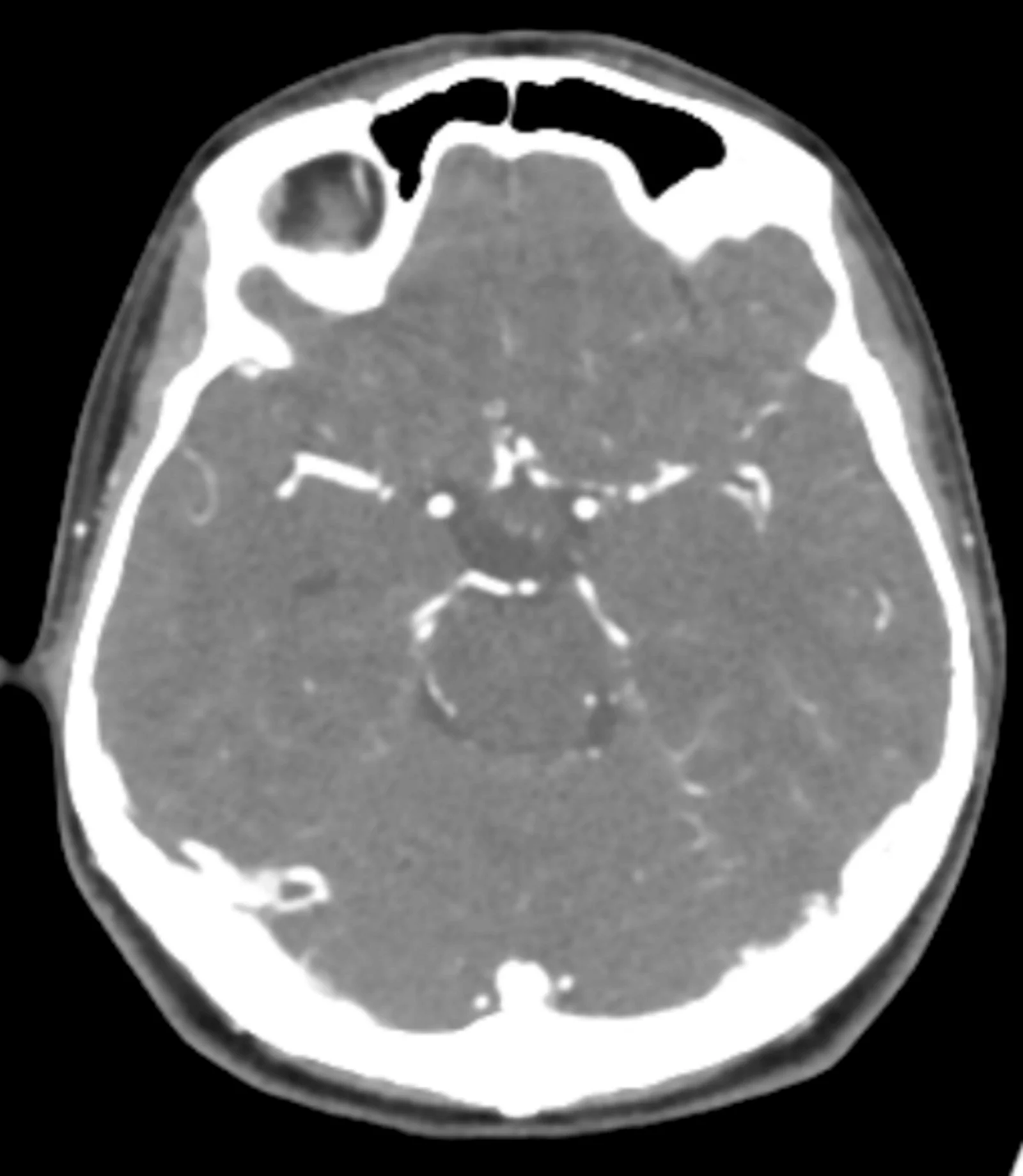
CT angiogram intracranial Normal morphology of the circle of Willis is seen. No aneurysm or dilatation was observed. CT: computed tomography

**Figure 4:**
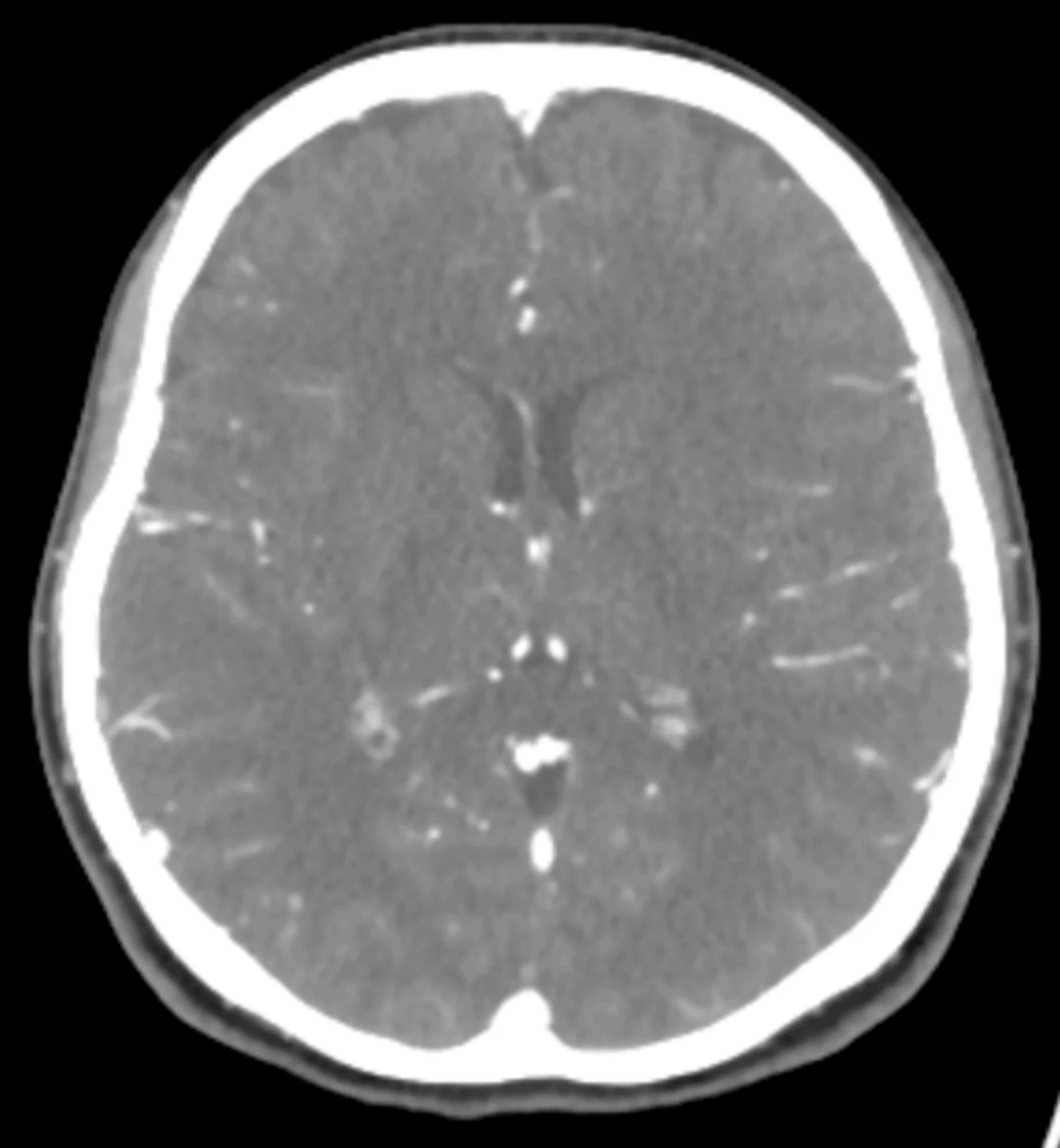
CT carotid and intracranial angiogram Normal opacification of the internal carotid arteries bilaterally is seen. No significant stenosis or occlusion was seen. CT: computed tomography

The initial plan was to correct the anemia and low blood pressure, for which she received two doses of iron infusion and intravenous fluids; however, symptoms persisted despite blood pressure correction and a hemoglobin increase to 92 g/L. 

For this reason, MRI of the brain with contrast was performed, which demonstrated smooth diffuse pachymeningeal enhancement over the cerebral and cerebellar convexities, prominence of the dural venous sinuses, and a full pituitary gland, without significant brain sagging (Figures 5-6).

**Figure 5:**
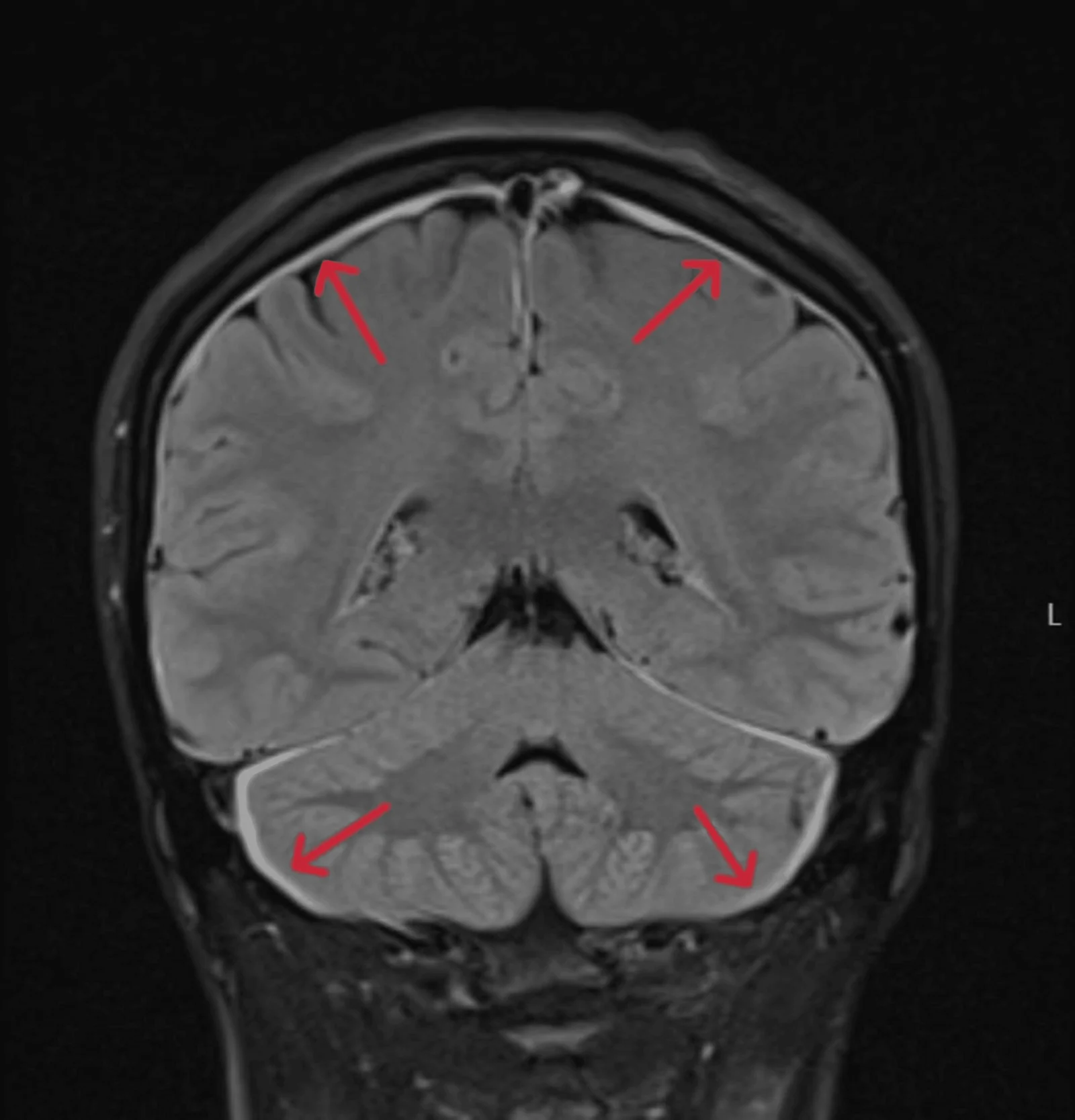
MRI brain Smooth pachymeningeal enhancement over the cerebral and cerebellar convexities is seen (arrows). MRI: magnetic resonance imaging

**Figure 6:**
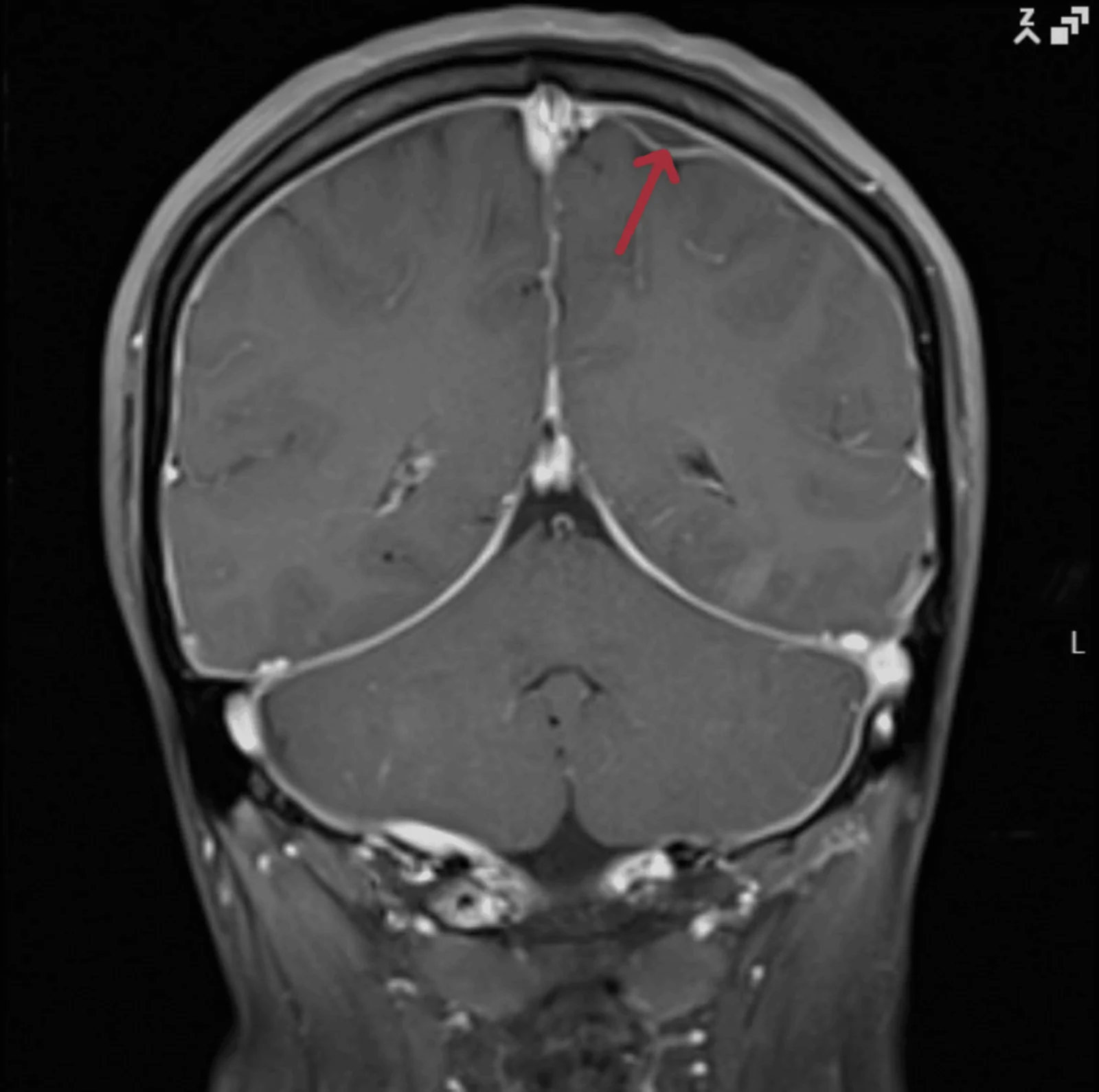
MRI brain and cervical spine Spinal imaging shows an epidural collection (arrow) that starts from the lower cervical canals. MRI: magnetic resonance imaging

These findings are characteristic of intracranial hypotension [8]. Fluid-attenuated inversion recovery (FLAIR) sequencing MRI of the whole spine revealed an extensive spinal longitudinal epidural CSF collection extending from the lower cervical spine to the lumbar region, with associated spinal pachymeningeal enhancement (Figures 7-8). This is clearly demonstrated on axial T2 images of the cervical spine and thoracic spine. Such spinal CSF collections are a recognized imaging feature of SIH [10]. No definite dural tear was identified, although a midline bony spur at the T1-T2 level was noted as a possible site of dural compromise, consistent with previously described mechanisms of spontaneous dural weakness [12]. The radiologist advised performing a CT cisternogram to identify the site of leakage; however, the neurosurgical team did not perform it. Therefore, the proposed association and mechanism remain speculative. She was accepted for neurosurgical transfer to a tertiary neurosurgical centre at Salford Hospital for further investigation.

**Figure 7:**
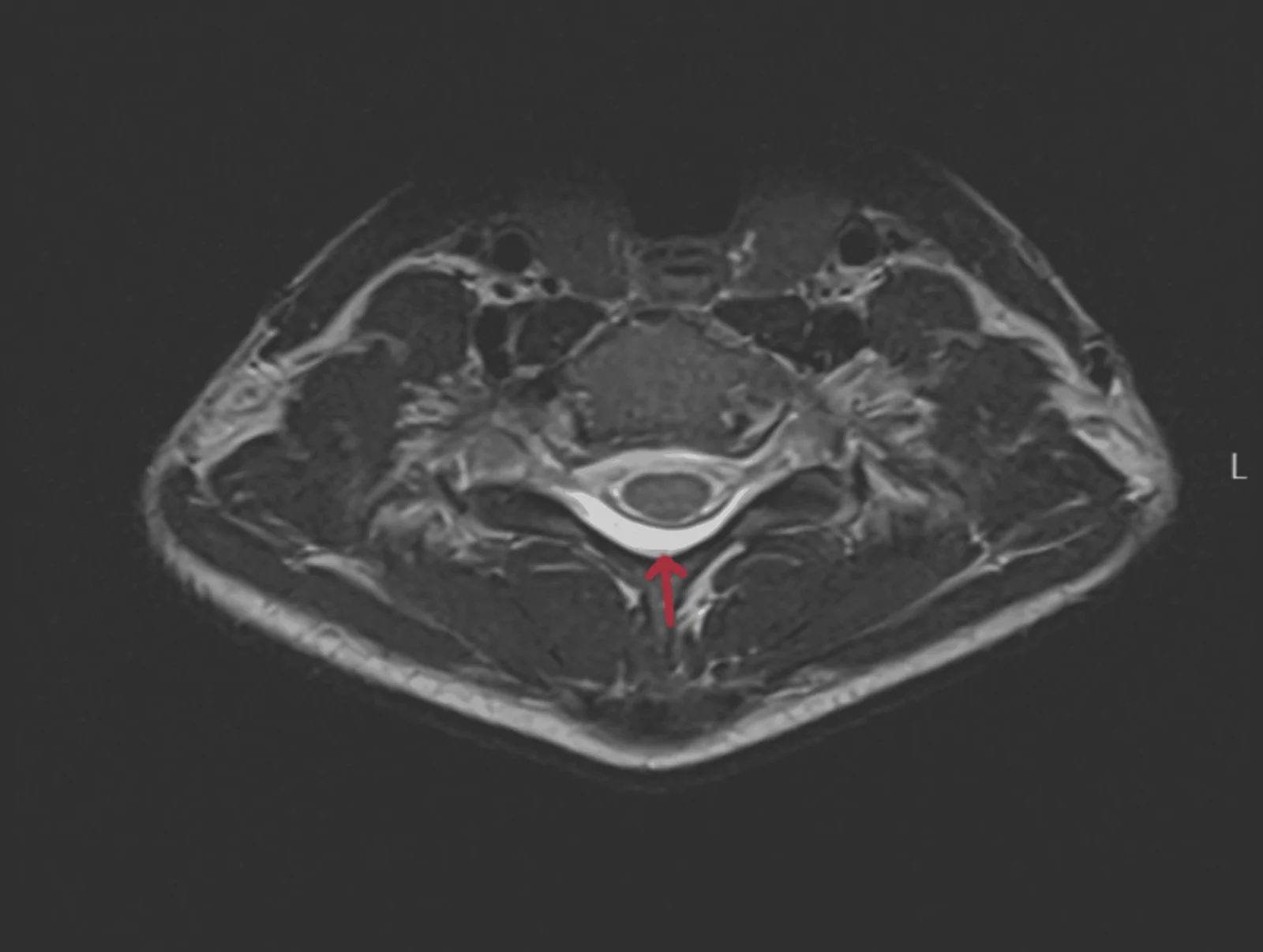
MRI spine Spinal imaging shows a clear spinal longitudinal epidural collection (arrow) that extends from the lower cervical canal all the way down into the lumbar canal. MRI: magnetic resonance imaging

**Figure 8:**
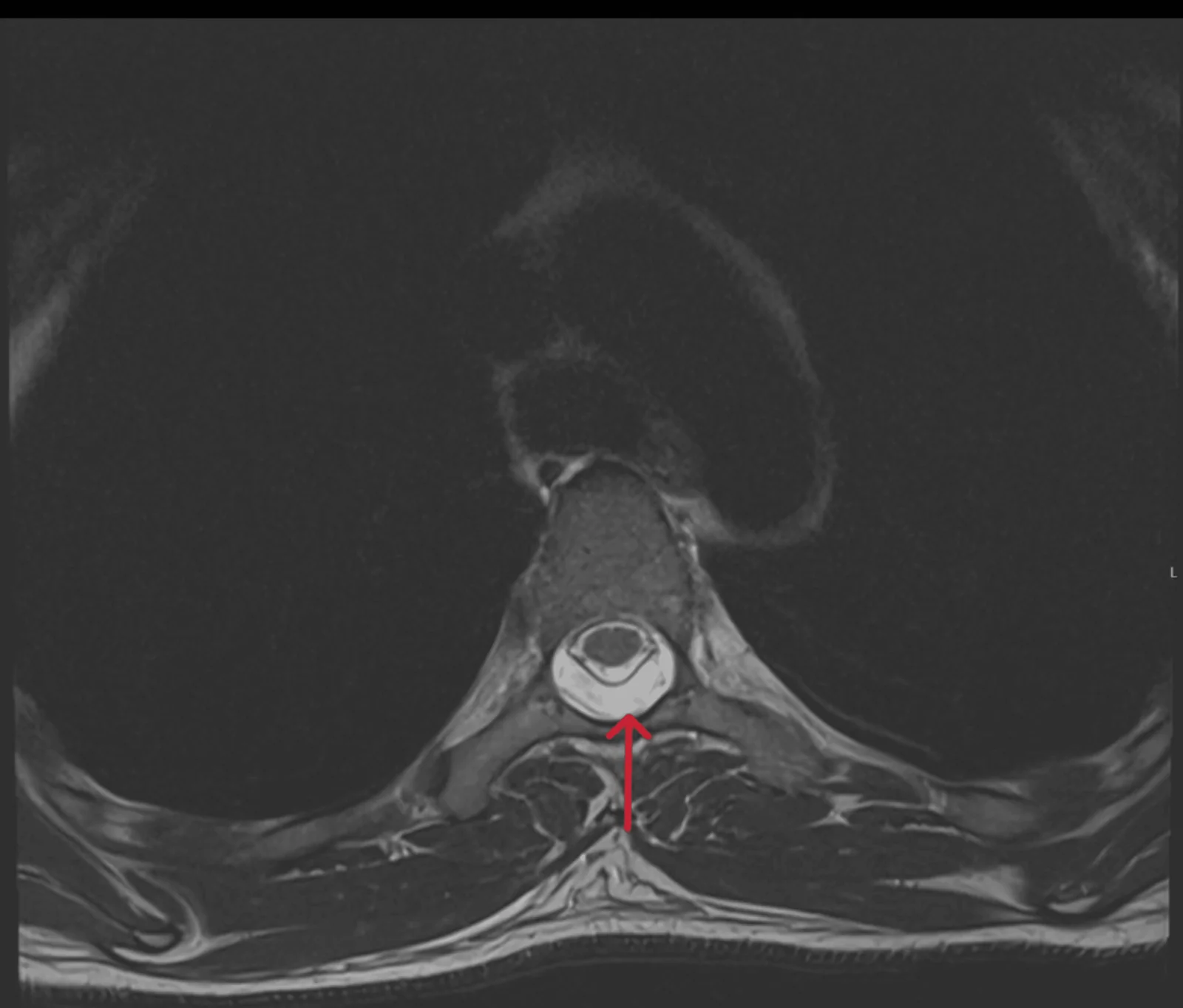
MRI spine Pachymeningeal enhancement (arrow) is observed in the spinal canal. MRI: magnetic resonance imaging

Although anemia and systemic hypotension may have contributed to the dizziness, presyncope, blurred vision, and lethargy, these findings could not explain the characteristic orthostatic headache or MRI abnormalities. 

Given the persistent orthostatic symptoms and MRI findings consistent with SIH, neurology and neurosurgery were consulted. She was accepted for neurosurgical transfer to a tertiary neurosurgical centre for further investigation, including consideration of CT myelography and definitive management of the CSF leak.

At the time of transfer, the patient remained symptomatic with headache, neck pain and dizziness on sitting upright. A non-targeted epidural patch was performed after transfer on day 2 of admission to the neurosurgical unit.

The patient's symptoms improved significantly on day 3 of admission to the neurosurgical unit, and she was discharged with planned follow-up imaging and intervention under specialist care.

## Discussion

SIH is a rare condition caused by a spinal CSF leak [1], with an estimated annual incidence of roughly 5 per 100,000 people [2]. It affects women twice as often as men and most frequently strikes middle-aged adults around 40 years old [2].

CSF leaks have been classified into four types according to their morphology and distance from the midline [13].

Type 1 leaks result from degenerative disc disease, which produces a mechanical tear in the ventral dura. Ventral dural tears account for a substantial proportion of patients with SIH, representing 48% of patients. On MRI, all patients with type 1 leaks demonstrate a spinal longitudinal extradural CSF collection (SLEC-P), regardless of whether intracranial abnormalities are present. Dynamic digital subtraction myelography (DSM) or dynamic CT myelography typically shows a characteristic ventral defect in the thecal sac. In some cases, the leak is associated with a calcific “microspur” arising from a degenerated disc. Dynamic imaging is essential because it captures the initial passage of contrast material along the spine. Without this technique, contrast mixing within the intradural and extradural spaces above and below the dural defect may obscure the precise site of leakage [13].

Type 2 leaks were also associated with SLEC-P in all cases. These leaks arise from a more laterally located dural tear and were a less frequent cause of SLEC-P. The associated extradural CSF collections may be located closer to the neural foramina and are not typically linked to degenerative disc disease. They are thought to originate at the base of the nerve root, where a pre-existing area of dural thinning or dehiscence allows CSF to escape. These lesions appeared to respond more favorably to repeated epidural blood patches (EBPs) and bed rest, potentially because the tears were not maintained by a disc protrusion or continuous flow, as occurs with a CSF-venous fistula (CVF) [13].

The recognition of CSF-venous fistulas (CVFs) by Schievink et al. represented a major advance in the management of SIH. It has been proposed that the dural defect responsible for a CVF develops farther along the nerve-root sleeve, near an arachnoid granulation or an area where the sleeve lies adjacent to a venous plexus. In this setting, extravasated CSF may enter a nearby venous channel. An embryonic pseudomeningocele may initially extend into the venous system and subsequently heal in an abnormal manner, producing a one-way CVF, which is classified as a type 3 CSF leak. This mechanism may explain why CVFs are often, although not invariably, located near structures resembling diverticula. CVFs generally do not respond to non-directed EBP because they are situated beyond the epidural space. The effectiveness of targeted regional EBP remains uncertain. Although CT-guided focal fibrin patching has been successful in some reports, it did not provide a durable cure in the authors’ experience. Surgical ligation, as described by Schievink et al., was considered the most effective treatment for these lesions [14].

Type 4 leaks were the least common variety observed. They arise from a distal nerve-root sleeve and do not enter the venous system. Instead, the leaked CSF tracks into and disperses through the adjacent fascial planes. Because of their distal location, type 4 leaks present as SLEC-N rather than SLEC-P and are not expected to respond to EBP [13].

The Bern score is a 9-point probabilistic grading system derived from brain MRI findings, aimed at predicting the likelihood of identifying an underlying spinal CSF leak in patients suspected of having SIH (Table 2). This tool was introduced in a 2019 study published in JAMA Neurology by Dobrocky et al. It incorporates three major and three minor neuroimaging criteria to assess a patient's probability of a leak, assisting specialists in deciding whether invasive spinal imaging is warranted. The system allocates points based on specific qualitative characteristics and quantitative measurements observed in a contrast-enhanced brain MRI [15].

**Table 2:** Bern score Score interpretation: 0 to 2 points indicate low probability (0% to 7% chance of a CSF leak); 3 to 4 points indicate intermediate probability; 5 to 9 points indicate high probability (67% to 100% chance of a CSF leak). CSF: cerebrospinal fluid

Major criteria (2 points each)
Pachymeningeal enhancement
Engorgement of venous sinus
Suprasellar cistern 4.0 mm or less
Minor criteria (1 point each)
Subdural fluid collection
Prepontine cistern 5.0 mm or less
Mamillopontine distance 6.5 mm or less

Despite the availability of MRI, its non-invasive nature, and the absence of ionizing radiation, it presents several drawbacks, including reduced sensitivity to low-flow leaks, an inability to identify subtle bone pathologies, and the presence of technical artifacts. Nonetheless, accurately pinpointing the location of the leak becomes difficult when a significant epidural collection is present. The intrathecal use of gadolinium-based contrast agents can enhance sensitivity, but an overdose of the contrast agent may result in neurological damage, and there is a lack of long-term safety data [16].

Following the detection of the epidural collection through MRI or CT myelography, further imaging techniques such as dynamic myelography, digital subtraction myelography, or dynamic CT myelography, customized to the suspected site of leakage, enable accurate localization of the CSF leak or the CSF-venous fistula [17].

There are multiple treatment options available for SIH: conservative therapy, EBP, or surgical intervention [18]. Conservative therapy involves bed rest to alleviate orthostatic headaches and facilitate the natural closure of CSF leak, along with adequate hydration. The effectiveness of corticosteroids remains uncertain and may pose risks of side effects. Caffeine and theophylline are occasionally suggested to enhance CSF production, although evidence supporting their efficacy is insufficient. The overall success rate of conservative management is approximately 28%. Notably, 61% of patients continue to experience at least moderate headaches after six months, and 50% still report symptoms after two years. Additionally, due to often delayed diagnoses, patients typically have already undergone conservative treatment by the time they present for further care [18].

EBP is the most frequently utilized treatment for spinal CSF leaks that do not improve with conservative measures. The reported success rate varies between 36% and 90%. This procedure typically involves the injection of a 10-50 mL of autologous blood, which provides immediate relief through volume replacement. The prolonged effectiveness in sealing the leak is attributed to the interaction between the blood and pro-coagulant elements in the leaking CSF, resulting in clot formation [18].

Non-targeted epidural blood patch (EBP), also known as a blind patch, can be performed in the lumbar region or at the thoracolumbar junction when the exact leak site is unidentified. Post-procedure, patients are advised to rest in the Trendelenburg position for a minimum of eight hours to allow the blood to reach the CSF leak. If necessary, the procedure may be repeated up to two times, with at least a seven-day interval between each. Targeted EBP, which may involve the use of autologous blood and/or fibrin glue, can enhance the response rate and is primarily applied in the thoracic and upper cervical areas. This approach is typically employed after two or three unsuccessful blind lumbar EBPs [18].

Surgery is advised for patients who do not respond to EBP and have clearly defined CSF leaks. Treatment for nerve root sleeve diverticula involves clipping the root sleeve, repairing the dura, or using epidural packing. Surgical intervention for ventral leaks is more challenging. A posterior transdural approach facilitates the repair of the dural tear while also allowing for the removal of related osteophytes. Development of minimally invasive techniques is progressing rapidly. Additionally, surgery is necessary for the repair of CSF-venous fistulas, as EBP tends to be less effective in these situations [18].

Although the overall outlook is positive, frequent occurrences of delays and missed diagnoses exist. There are several reasons for that: First is atypical symptom presentation. Typically, a classic headache intensifies when the patient is upright and alleviates when lying down. However, many patients experience persistent, non-positional headaches or may prioritize symptoms such as neck pain, dizziness, or cognitive difficulties over time. Secondly, there is an overlap with common disorders, as symptoms often resemble those of primary conditions such as migraines, tension headaches, sinusitis, meningitis, or psychiatric disorders, which can lead healthcare providers to follow incorrect treatment routes. Thirdly, over 50% of the patients with confirmed spinal CSF leaks show a normal opening pressure during a lumbar puncture, which may lead some physicians to mistakenly rule out the diagnosis. Lastly, radiologists who are not specialized may overlook subtle indications of brain sagging or dural enhancement on standard brain MRI scans, or they may misinterpret these signs as other conditions such as pituitary tumors or inflammatory diseases. Despite limited knowledge of SIH among primary care providers and non-neurology specialists, which hinders timely referrals to specialists and the use of advanced spinal imaging, the outlook for SIH is quite favorable, with most patients achieving complete recovery either spontaneously or through specific interventions such as an EBP [19].

It has been noted that patients who present with distinct abnormal findings on initial brain or spine MRI scans tend to have a more straightforward diagnostic and recovery process compared to those whose baseline imaging appears entirely normal. If the patient is not treated for extended periods, severe chronic cases may lead to serious secondary complications, including subdural hematomas, superficial siderosis, or reversible cognitive decline [20].

In some patients, symptoms may recur after initial successful treatment. Recurrences are generally linked to the ongoing presence of an unmasked or undetected primary structural weakness in the spinal dura, an untreated secondary leak site, or the specific mechanics of complex lesions such as the CSF-venous fistulas. Many cases of recurrence or persistence are effectively managed with repeat or more accurately targeted EBPs or specialized surgical repair [20].

A negative brain MRI indicating no downward brain displacement is significant in cases of SIH because it demonstrates that normal imaging does not exclude the possibility of a spinal CSF leak. Up to one-third or more of patients with verified CSF leaks and typical orthostatic headaches may present with a normal brain MRI, showing neither downward displacement nor pachymeningeal enhancement. Clinicians may incorrectly conclude that "no brain sag equates to no leak," which can result in harmful delays in diagnosis for patients enduring severe symptoms. This condition is primarily caused by a reduction in CSF volume (hypovolemia) rather than merely a specific low-pressure threshold or anatomical sagging. A patient may not exhibit brain sag yet still suffer from significant symptoms or cognitive alterations due to localized mechanical strain. If a patient reports classic postural headaches (aggravated when upright and alleviated by lying down), additional diagnostic evaluations along the spinal axis, such as myelography-CT or focused dynamic imaging, are essential, even in the presence of a negative cranial MRI [21].

This case illustrates a classic but often overlooked presentation of SIH [1-7]. The hallmark orthostatic headache, accompanied by neck pain, dizziness, and vomiting, was initially non-specific and occurred in the absence of trauma or neurological deficit, a recognised diagnostic pitfall [1].

Lumbar puncture was deliberately avoided in this patient because of the suspected spinal CSF leak and the imaging was considered sufficient.

MRI findings of diffuse pachymeningeal enhancement and an extensive spinal epidural CSF collection were key to establishing the diagnosis in this case [8-10].

SIH is thought to result from underlying dural weakness, leading to spontaneous CSF leakage and reduced intracranial CSF volume [1]. Prompt diagnosis is essential, as untreated SIH may lead to complications such as subdural effusions or hemorrhage [11]. This case emphasizes the importance of considering SIH early in patients presenting with acute orthostatic headache and neck pain, even when initial CT imaging is normal, to prevent early complications.

## Conclusions

We endeavor to enhance recognition of SIH among neurologists and other healthcare practitioners who encounter patients presenting with headache complaints. Accurate diagnosis depends on heightened clinical vigilance and appropriate interpretation of the expanding array of clinical manifestations and findings that should prompt radiological investigation, with contrast-enhanced MRI of the cranium being the preferred modality, as normal CT imaging does not exclude SIH. The presence of diffuse, uniform dural enhancement on cerebral MRI is highly indicative of SIH and should promptly be followed by spinal imaging studies to identify the CSF fluid leak site. Extensive spinal CSF leaks may occur without an identifiable dural defect on initial imaging. While conservative therapeutic approaches represent the initial management strategy, such interventions frequently prove inadequate in symptom resolution, requiring early patient referral to neurosurgical specialists to prevent complications and guide definitive management.

The diagnosis of SIH requires a high degree of clinical suspicion and should be considered in patients presenting with acute orthostatic headache and neck pain without preceding trauma. Our patient did complain of headache worsening in the upright position, which was initially labeled as related to anemia and low blood pressure, suspicions that delayed the correct diagnosis and treatment for nearly 10 days. Once the patient received the appropriate treatment, she successfully returned to occupational and daily activities.
